# An infrared photothermoelectric detector enabled by MXene and PEDOT:PSS composite for noncontact fingertip tracking

**DOI:** 10.1038/s41378-022-00454-3

**Published:** 2023-02-27

**Authors:** Jiaqi Wang, Zhemiao Xie, Guanxuan Lu, Jiayu Alexander Liu, John T. W. Yeow

**Affiliations:** Advanced Micro-/Nano- Devices Lab, Department of Systems Design Engineering, University of Waterloo, 200 University Ave West, Waterloo, ON N2L 3G1 Canada

**Keywords:** Electrical and electronic engineering, Electronic properties and materials

## Abstract

Photothermoelectric (PTE) detectors functioning on the infrared spectrum show much potential for use in many fields, such as energy harvesting, nondestructive monitoring, and imaging fields. Recent advances in low-dimensional and semiconductor materials research have facilitated new opportunities for PTE detectors to be applied in material and structural design. However, these materials applied in PTE detectors face some challenges, such as unstable properties, high infrared reflection, and miniaturization issues. Herein, we report our fabrication of scalable bias-free PTE detectors based on Ti_3_C_2_ and poly(3,4-ethylenedioxythiophene):polystyrene sulfonate (PEDOT:PSS) composites and characterization of their composite morphology and broadband photoresponse. We also discuss various PTE engineering strategies, including substrate choices, electrode types, deposition methods, and vacuum conditions. Furthermore, we simulate metamaterials using different materials and hole sizes and fabricated a gold metamaterial with a bottom-up configuration by simultaneously combining MXene and polymer, which achieved an infrared photoresponse enhancement. Finally, we demonstrate a fingertip gesture response using the metamaterial-integrated PTE detector. This research proposes numerous implications of MXene and its related composites for wearable devices and Internet of Things (IoT) applications, such as the continuous biomedical tracking of human health conditions.

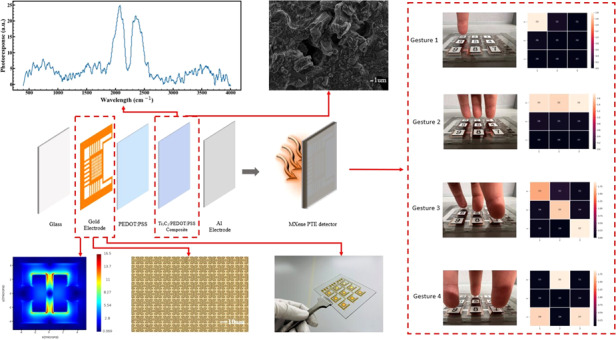

## Introduction

Mid-infrared (MIR, 2.5–25 μm) detectors that can convert MIR radiation into electrical signals have been employed in many fields^1^, such as in digital imaging^2^, wearable devices^3^, and energy-harvesting fields^4,5^. Presently, commercial MIR detectors mainly adopt traditional mercury cadmium telluride (MCT)^6^, gallium arsenide (GaAs)^7^, indium antimonide (InSb)^8^, or other quantum materials^9,10^. However, most of these detectors generally suffer from issues such as material toxicity, cryogenic operating condition requirements, strong bandgap dependence, and high-cost fabrication processes^11^. In contrast, photothermoelectric (PTE) detectors combining photothermal and thermoelectric conversion can avoid bandgap dependence and achieve broadband infrared detection at room temperature without external bias^12^. Previously, cadmium sulfide (CdS) crystals^13^ or GaAs semiconductors^14^ were selected as traditional materials. Unfortunately, their PTE responses are relatively weak, and thus novel PTE materials are urgently needed.

In the last decade, a wide range of PTE materials have been comprehensively reported, such as carbon nanotubes (CNTs)^15,16^, graphene^17,18^, and black phosphorus^19^. With high carrier mobility and scalable detection, the emergence of these low-dimensional materials provides guidelines for the miniaturization of on-chip design. However, there are still some significant limitations. For single-layer graphene materials, their gapless band structures endow them with excellent optical properties, but their fabrication processes are comparatively complex and costly^20,21^. Black phosphorus also shows competitive PTE performance, but its unstable chemical properties make it challenging to use for practical applications^22,23^. Various CNTs, including single-walled CNTs, multiwalled CNTs, and CNT-based composites, have been demonstrated to be PTE materials with advantageous photoresponses^16,24–26^. However, the armchair or zig–zag directions of CNTs entail complex growth methods and accurate control, which hinders their further development^27^. Our group has summarized the recent progress of PTE materials and proposed the potential of using MXenes for PTE detectors^28^. MXenes, as a flourishing group of two-dimensional materials, were initially discovered by Yury Gogotsi’s team at Drexel University in 2011^29^. This material family is composed of transition metal nitrides, carbides, and carbonitrides. Generally, the MXene formula is defined as M_n+1_X_n_T_x_, where M expresses the transition metal, X indicates carbon or nitrogen sites, and T expresses the surface terminations of outer transition metal layers^30^. MXenes have great application potential in photodetectors and surface resonance sensors^31–33^, which exhibit advantageous optoelectrical properties, such as broadband electromagnetic absorption and surface plasma excitation^34–36^.

Composites based on a polymer matrix and low-dimensional nanofillers are advantageous because of their facile fabrication processes. For the matrix, poly(3,4-ethylenedioxythiophene):polystyrene sulfonate (PEDOT:PSS) has been verified to have the highest thermoelectric conversion efficiency, with ZT = 0.42^37^. It has also been used with other low-dimensional materials to act as an excellent PTE-active composite matrix^38,39^. In 2016, it was indicated that pristine MXene and MXene-polymer composite films produced an excellent electromagnetic shielding (EMS) effect, demonstrating their photon absorption ability^40^. Furthermore, as a state-of-the-art EMS material, multiple reflections and absorption mechanisms convert the absorbed photons into thermal energy inside the MXene material. Then, in 2017, inspired by the abovementioned electromagnetic wave absorption, Li et al. substantiated MXene’s light-to-heat conversion ability of 100%^41^. In 2020, Guan et al. studied the thermoelectric properties of MXene and polymer composite membranes^42^. By incorporating the n-type Ti_3_C_2_T_x_ into PEDOT:PSS, the Seebeck coefficient of the composite increased from 23 to 57.3 μV/K^42^. Therefore, MXene and polymer composite materials should be ideal candidates for broadband PTE detectors.

In this research, we propose a scalable sensitive MIR PTE detector utilizing an MXene/PEDOT:PSS composite. Due to the strong absorption and multiple reflection mechanism of MXene, the PTE performance is also enhanced. A broadband photoresponse with a varying spectral range from 2.5 μm to 25 μm and a peak responsivity of 0.12 V/W at 4.5 μm are measured for the MXene-based PTE detector. We investigate many PTE engineering strategies and optimization methods, such as electrode choices and vacuum conditions. We also integrate a complementary split ring resonator metamaterial into the composite PTE detector. The detector shows a photocurrent enhancement and characteristic noncontact fingertip radiation response measurements, which offers the potential for future realistic health monitoring.

## Results and discussion

### Device fabrication and structural characterization

**Fig. 1:**
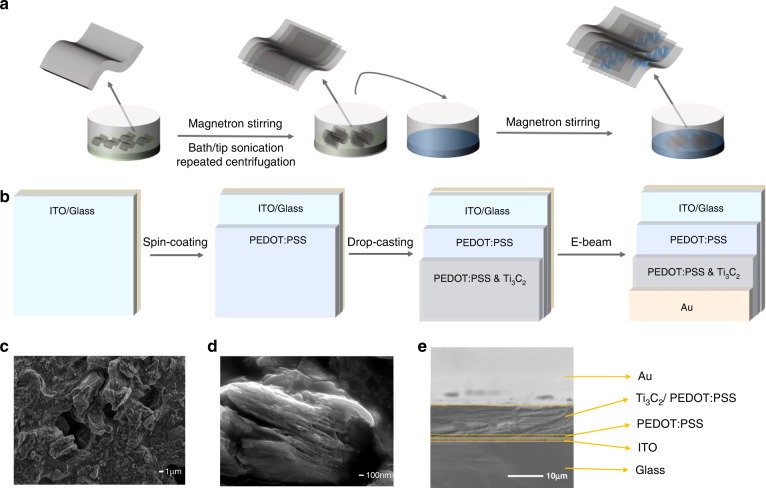
Design and fabrication of the MXene/PEDOT:PSS PTE detector. **a** Preparation of Ti_3_C_2_/PEDOT:PSS composite solution. Gray, green, and blue colors represent MXene, DMSO, and PEDOT:PSS, respectively. **b** Fabrication process of the Au/(Ti_3_C_2_/PEDOT:PSS)@PEDOT:PSS/ITO structure on the glass substrate. **c** SEM imaging of the Ti_3_C_2_/PEDOT:PSS membrane. The scale bar is 1.0 μm. **d** SEM imaging of delaminated Ti_3_C_2_ flakes with gaps. The scale bar is 100 nm. **e** Cross-sectional SEM imaging of a single-pixel PTE detector. The scale bar is 10 μm

We combined sonication and solvent exchange methods to synthesize delaminated MXene (d-MXene) (Fig. 1a)^43^. After dispersing the MXene powder into polar dimethyl sulfoxide (DMSO) solution, both bath sonication and tip sonication were required to delaminate MXene. The control of sonication time and power will be discussed in a later section. Repeated centrifugation by removing the supernatant and adding DI water helped maintain the stability of MXene. Different MXene solutions with different repeated centrifugation can be observed in Supplementary Fig. S1. The pristine Ti_3_C_2_ colloidal solution dispersed well in DI water by the Tyndall scattering phenomenon, as shown in Supplementary Fig. S2b. By mixing dispersed d-MXene and PEDOT:PSS followed by overnight magnetron stirring, we obtained a stable Ti_3_C_2_/PEDOT:PSS solution. The solution was stored under N_2_ conditions for further film coating. The fabrication process of the vertical MXene detector and array configuration is illustrated in Fig. 1b. A thin PEDOT:PSS thin film was spin-coated to the top of the indium tin oxide (ITO) bottom electrode. This thin film increased the adhesion of MXene-based composite materials and reduced the chances of peel-off. Then, a thick Ti_3_C_2_/PEDOT:PSS membrane was deposited by a facile drop-casting method.

Despite the resolution limitations of SEM, the micrometer-/nanometer-level pores were tracked in detail, but many voids were seen among the internal MXene flakes (Fig. 1c). This mesoporous structure helps construct the conductive percolation path, which is advantageous for carriers and ionic transportation^44^. In addition, the small flakes are beneficial to the mesoporous structures. The hybrid interaction between Ti_3_C_2_T_x_ and PEDOT:PSS is illustrated in Fig. 1c. T_x_, such as –OH, = O, and –F, facilitates connections with polar groups^40^, and the quinoid configuration is formed. This means that the introduction of PEDOT:PSS facilitates the interconnection of MXene flakes (Fig. 1d).

### PTE mechanism

In this section, we discuss the PTE mechanism of this vertical MXene-based detector (Fig. 1e). The PTE mechanism includes photothermal and thermoelectric conversion. When the radiation illuminates the thin gold top electrode with a thickness of 25 nm under the skin limit, most of the illumination passes through the electrodes^45^. Due to electron excitation, the top gold electrode is thermalized and acts as the heat source. Allowing for the lamellar structure of MXene and the polarization of termination groups, such as OH, F, and S^40^, the electromagnetic radiation is continuously reflected and eventually absorbed within the MXene/PEDOT:PSS composite membrane. PEDOT:PSS with coagulated structures results in a rough composite membrane morphology and connects the MXene flakes, which reduces the resistivity across the MXene flakes. In addition, the ITO electrode and glass substrate serve as the heat sink. Thus, in this vertical configuration, the temperature difference forms vertically. The infrared radiation-induced thermoelectric voltage can be expressed as

$$\begin{array}{ll} V &= {\mathop {\int}_{ITO}^{Au} S\left( z \right)\nabla T\left( z \right)dz}\\ &= \mathop {\int }\nolimits_{ITO}^{PEDOT:PSS} S\left( z \right)\nabla T\left( z \right)dz+ \mathop {\int }\nolimits_{PEDOT:PSS}^{Composite} S\left( z \right)\nabla T\left( z \right)dz\\& \quad\, +\, \mathop {\int }\nolimits_{Composite}^{Au} S\left( z \right)\nabla T\left( z \right)dz \end{array}$$

where S expresses the Seebeck coefficient and T represents the temperature. This composite material can achieve excellent conversion from photons into thermal or electrical energy, which further benefits optoelectrical devices and especially paves the path for PTE detectors.

### Optoelectrical measurement

**Fig. 2:**
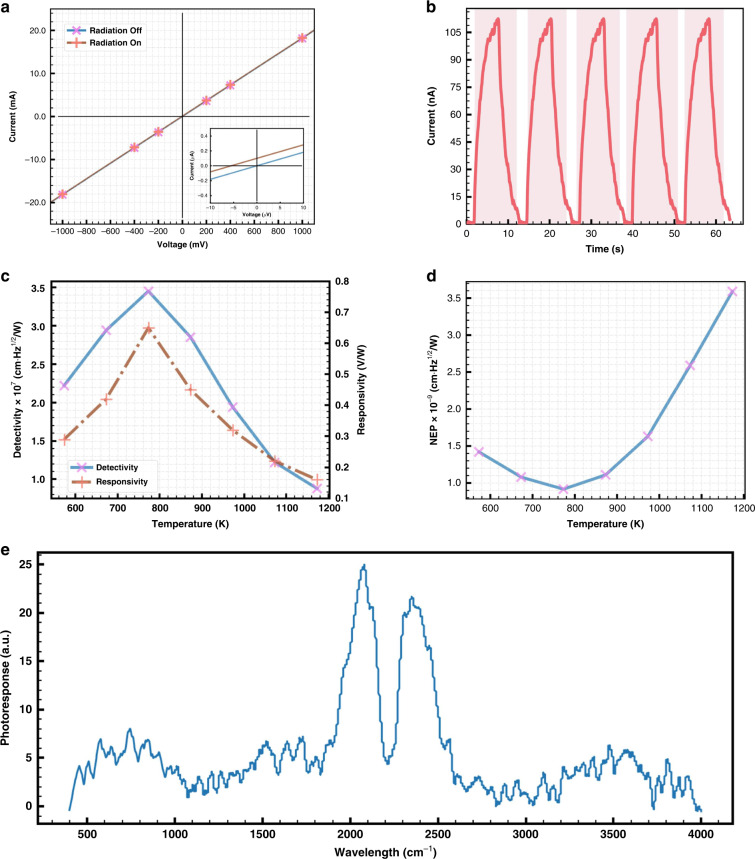
Optoelectrical characterization of a single-pixel PTE detector based on drop-cast Ti_3_C_2_/PEDOT:PSS. **a** I-V characteristics. **b** Response time. **c** Detectivity and responsivity of the detector under different blackbody radiation. **d** NEP dependence on the blackbody radiation temperature. **e** FTIR photocurrent measurement

The current–voltage (I-V) curves with or without blackbody infrared radiation are presented. The photocurrent is characterized at a blackbody temperature of 773 K without an external bias (Fig. 2a). The linear performance validates the ohmic contact between different layers. With the blackbody temperature at 773 K, when the irradiation is at on or off status, temporal responses exhibit excellent reproducibility for the composite PTE detector placed on the glass substrate (Fig. 2b). The detectivity that characterizes the ability of photon identification from ambient noise is expressed as $D^ \ast = \left( {\frac{V}{{P_i}}} \right)\left( {\frac{{\sqrt s }}{{V_t}}} \right) = R_V\left( {\frac{{\sqrt s }}{{V_t}}} \right)$, where V represents the photovoltage, P_i_ is the incident irradiation power, s is the active area, V_t_ is the thermal noise, and R_V_ expresses the responsivity^5^. The responsivity and detectivity that correlate with different blackbody radiation temperatures are shown in Fig. 2c. As the blackbody temperature increases from 573 to 773 K, the photon power density increases simultaneously. The detectivity and responsivity demonstrate a 50% increase. However, the subsequent increase in blackbody temperature causes a reduction in both detectivity and responsivity. The detectivity is ~10^7^ Jones, with a peak value of 3.5 × 10^7^ Jones at 2060 cm^−1^. The enhanced detectivity may contribute to the high infrared absorption and suppressed hot-carrier relaxation of the composite in this regime. Noise-equivalent power (NEP) is another term for characterizing the sensitivity of the PTE detector and can be defined as $NEP = \frac{{\sqrt {A\Delta f} }}{{D^ \ast }}$, where A is the photoactive area and Δ*f* is the bandwidth. As seen from Fig. 2d, the NEP first shows an increasing and then decreasing trend, which suits the changing trend of detectivity. This change may partly originate from different absorption spectra. The room-temperature FTIR photocurrent spectrum of the Ti_3_C_2_/PEDOT:PSS composite is plotted in Fig. 2e. In the PTE mechanism, the thermal Johnson–Nyquist noise level does not correlate with the spectrum. In addition, hot-carrier-assisted PTE conversion may exist in this vertical MXene composite detector. Due to the small electron thermal conductivity, the electron temperature will increase faster than the phonon temperature, and thus nonequilibrium temperature transfer may occur. The electron temperature difference induces electron flow. Furthermore, electron flow results in the generation of electron–hole pairs and strong interactions among electrons. The energy absorbed by the MXene composite warms up the carriers. In the two-dimensional material system, the interaction between electrons and phonons is significantly suppressed, and thus, the hot carriers mainly facilitate the PTE conversion. By optimizing the material design, this vertical detector’s response time may be significantly enhanced.

In addition, a thin semitransparent Ti_3_C_2_/PEDOT:PSS film can be obtained by utilizing the spin-coating method. By combining two transparent ITO electrodes and a spin-coated composite thin film, the whole device is configured as ITO/(Ti_3_C_2_/PEDOT:PSS)@PEDOT:PSS/ITO. The photographic image and UV‒Vis transmittance spectrum are shown in Supplementary Fig. S3. In a low-dimensional material system, flake agglomeration is a major issue, which causes nonuniformity and further hinders transparency enhancement. A reasonable mitigation solution is lowering the speed of spin coating. In addition, additional substrate cleaning steps, such as ozone addressing, are beneficial.

### PTE engineering strategies

**Fig. 3:**
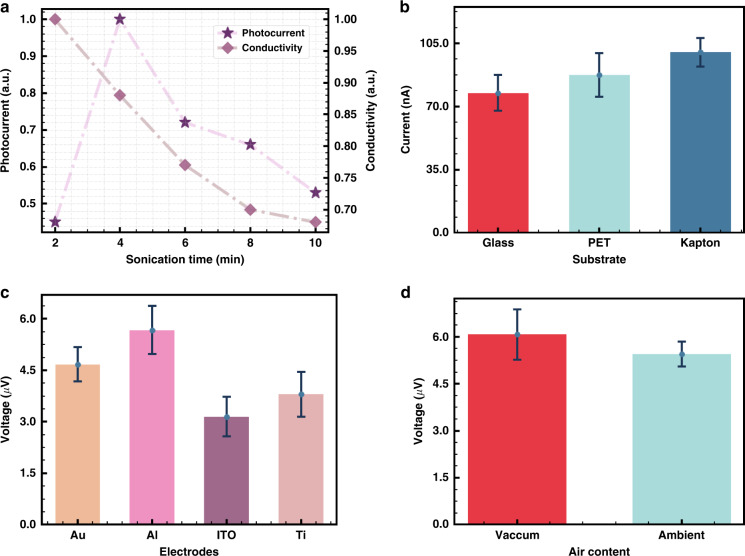
Engineering optimization strategies of the vertical PTE detector. **a** Sonication time. **b** Substrate material. **c** Top electrode material. **d** Vacuum conditions

As the micro/nanofiller in this composite, the MXene flake size may affect the optoelectrical properties and even the PTE effect. As shown in Supplementary Fig. S4, tip sonication can cut the MXene flake into small pieces. According to the modified sonication time, the MXene size also changes, which matches previous research results^42^. With increasing sonication time, the MXene size decreases, and conductive path damage occurs between MXene and the polymer (Fig. 3a). Although the conductive network is easier to form, the resistivity inevitably increases. Overall, excellent thermoelectric properties or PTE effects originate from proper MXene size, PEDOT:PSS concentration, and their coupling effect^42^. However, high sonication power may cause localized thermal accumulation, damaging the bonding interaction between PEDOT and PSS and further hindering the conductivity and PTE response (Supplementary Fig. S5).

The substrate experiment was also carried out at room temperature, and the top of the substrate was selected as the ITO/(MXene/PEDOT:PSS)/PEDOT:PSS/ITO structure. Notably, thermal dissipation occurs between the active layer and substrate, and thus, the thermal conductivity of the substrate is critical. As seen in Fig. 3b, polyimide shows the highest response in the substrate-based device because it shows the lowest thermal conductivity (*k* = 0.12 W/mK) compared with glass (*k* = 1.05 W/mK) and polyethylene terephthalate (PET, *k* = 0.3 W/mK).

We then prepare four top electrode materials, including ITO, Al, Ti, and Au. In this optimization design, the thickness of the electrode is controlled down to 20 nm. The reason for selecting this value refers to the skin limit. The electrode thickness of 20 nm is smaller than the skin limit in the infrared spectrum. The photoresponse ranking of these matched materials is Al>Au≈Ti>ITO (Fig. 3c), which matches the previous results^45^. Al shows the best photoresponse, but the Al electrode is prone to oxidization, which influences the conductivity and even the PTE response. Although gold shows a relatively weaker photoresponse than Al, it can be designed as a metamaterial^46^. Ti can demonstrate better adhesion with the substrate and photoactive layer because the photoactive layer Ti_3_C_2_T_x_ is also an enriched Ti-element material. In contrast, ITO can provide transparent properties, which benefits other wearable devices or the Internet of Things (IoT). Therefore, a trade-off is required when we adopt electrode materials.

Furthermore, we adopt E-beam and magnetic sputtering as two methods to deposit 20 nm electrodes (Supplementary Fig. S6). The device using sputtering strategies shows ~5–15% higher performance than that using E-beam deposition. Generally, magnetic sputtering can demonstrate a higher uniformity and film density than E-beams, which further affects the mechanical, electrical, and thermal contacts^47^, eventually improving the performance of PTE detectors.

Given that the PTE effect is a thermal-based mechanism, we investigate the influence of ambient air contents on the detector photoresponse. We create two different conditions, atmospheric and high vacuum (Supplementary Fig. S7). The blackbody radiation source is set up at 773 K and ~20 cm away from the detector. As seen in Fig. 3d, the photocurrent increases by 20%, and only a small variation in the response time appears. This may originate from the reduced thermal loss to the environment, i.e., more energy can be converted from light energy into heat, which improves the first-step conversion efficiency of the PTE effect. This vacuum-enhanced photoresponse helps maintain long-term stability. Furthermore, allowing for the configuration of the PTE detector and controlling the vacuum conditions of different components, such as electrodes and channels, may benefit the performance of the PTE detector.

In addition, we also study the illumination position dependence on the PTE response. This experiment was performed under ambient conditions at room temperature. The results can be understood according to previous research theory^24,48^. Within the PTE configuration, the PTE response induced by radiation at different positions is given by PTE = (*S*_1_ − *S*_2_)(*T*_1_ − *T*_2_), where *S*_1_ or *S*_2_ represents the Seebeck coefficients of adjacent layers, and *T*_1_ or *T*_2_ represents the real-time temperature. Supplementary Fig. S8 shows the photocurrent performance at different positions. The results show that the PTE response mainly occurs at the interface. The difference in Seebeck coefficients between adjacent material interfaces plays an important role in the PTE response. The interfaces between PEDOT:PSS and the composite show ~30.0 μV/K and a 0.1 K temperature difference, resulting in a 3.0 μV photovoltage. The photovoltage between the two interfaces exhibits an evident reduction.

### Metamaterials design

**Fig. 4:**
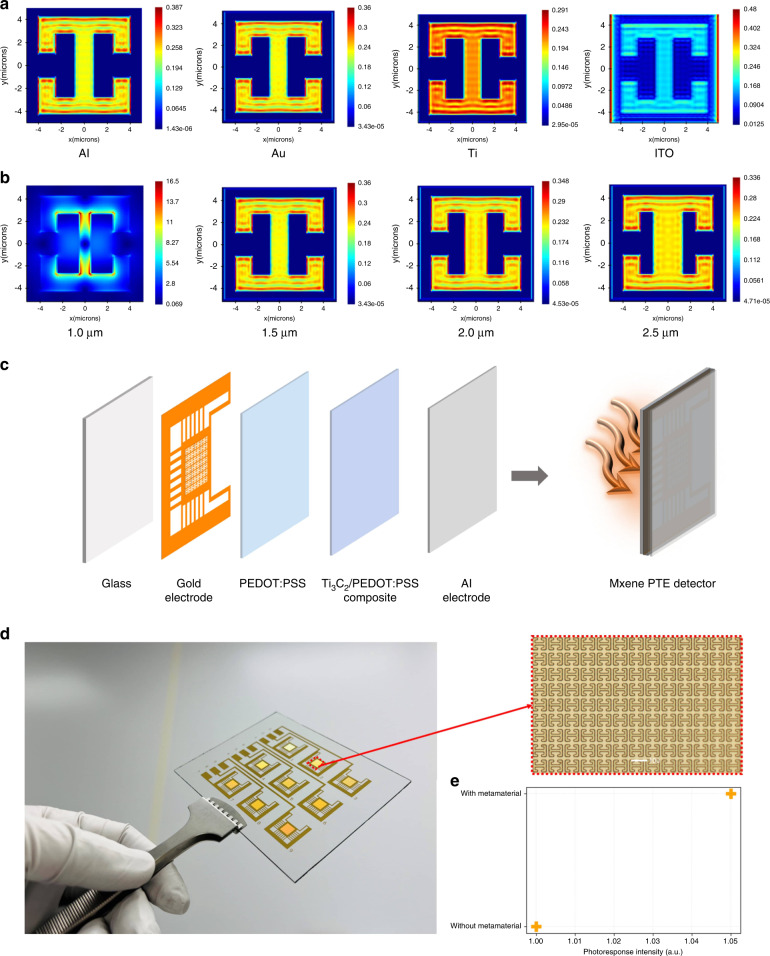
Metamaterial design. **a** Electromagnetic simulation results using Al, Au, Ti, and ITO. **b** Electromagnetic simulation results with different hole sizes of 1.0, 1.5, 2.0, and 2.5 μm. The metamaterial is gold. **c** Fabrication process of the PTE detector integrated with metamaterial design. **d** Optical imaging of metamaterials on glass. The red square is the enlarged photographic metamaterial imaging. **e** Photocurrent enhancement with or without metamaterial

Metamaterials design, as a method of improving electromagnetic absorption, can play an important role in designing PTE detectors. An optical simulation is carried out by using Lumerical software, and the details of one metamaterial unit are listed in Supplementary Fig. S9 and Supplementary Table S1. The simulated MIR radiation will illuminate from the glass direction. We simulate different metamaterials, including Al, Au, Ti, and ITO (Fig. 4a). All the electromagnetic field intensities using these four materials improve. Thus, in specific applications, we need to consider other factors, such as fabrication processes that are compatible with typical metal materials. In addition, Al and Ti may be easily oxidized, but they are inexpensive. The price of Au is expensive, but its conductivity is higher than others. ITO can show high transparency. By using the gold metamaterial, we also simulate different channel sizes, including 1.0, 1.5, 2.0, and 2.5 μm. A simulated electromagnetic enhancement can be seen in Fig. 4b. Although the metamaterial with a 1.0 μm channel size shows the strongest field enhancement, maskless alignment (MLA) usually shows a round edge, and pattern resolution degrades considerably. Thus, we choose a channel width of 1.5 μm. Given that the micrometer-level pattern is challenging to fabricate after depositing MXene/PEDOT:PSS film, we utilize a bottom-up strategy to fabricate this PTE detector (Fig. 4c), and the blackbody radiation will illuminate from the glass direction. Supplementary Fig. S10 shows the cross-sectional SEM image of this metamaterial-integrated device. Next, we describe the fabrication process of the metamaterial. A gold metamaterial structure is fabricated on the top of the glass substrate by MLA, E-beam deposition, and a lift-off process. Then, PEDOT:PSS is spin-coated to improve the adhesion between the gold electrode and MXene composite. Next, the MXene/PEDOT:PSS composite is drop-cast on each pixel. Finally, Al electrodes are deposited, followed by aluminum wire bonding. Using this fabrication strategy, the metamaterial surface can be protected, and the PTE detector can achieve long-term stability. As seen from Fig. 4e, the measured responsivity can show a 5% enhancement by integrating this metamaterial structure.

### Applications

**Fig. 5:**
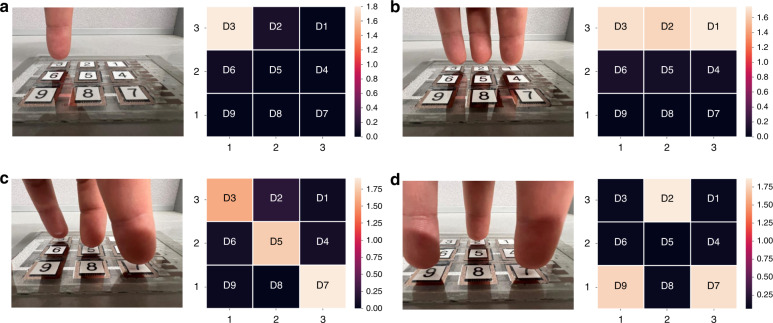
Fingertip tracking applications. **a**–**d** Fingertips are placed on the top of “D3”, “D3, D2, D1”, “D3, D5, D7”, and “D2, D7, D9”, respectively. The fingertips are placed 2 mm away from the glass layer. The color bar indicates the intensity of the photovoltage. The “D1”, “D2”, “D3”, “D4”, “D5”, “D6”, “D7”, “D8”, and “D9” labels match the device number from 1 to 9

Pandemic respiratory syndrome coronavirus 2 has had severe effects around the world. The causative agent of coronavirus disease can spread via respiratory droplets and close contact, such as through elevator buttons or restroom faucets^49^. Therefore, noncontact finger-sensing devices are urgently needed to prevent the spread of this novel coronavirus. Compared to direct contact sensors, self-powered novel infrared sensors can be controlled in a noncontact way through infrared response changes, thus avoiding mechanical touch and bacterial transmission. Importantly, infrared sensors can efficiently utilize human passive radiation to collect information and achieve relatively long-distance signal control, which would be a potential control method in advanced human‒machine interactions. In this research work, we tested the human fingertip response and recorded the photovoltage change of nine pixels by inserting a 2001-TCSCAN card into a Keithley DMM-6500 to achieve a multichannel measurement. As seen in Fig. 5a–d, we tested different positions and measured the fingertip radiation measurement. Different shapes demonstrated the noncontact radiation tracking viability of this detector.

## Conclusion

In conclusion, we presented a scalable broadband PTE detector based on MXene and a polymer composite and discussed its PTE mechanism. We also characterized the morphology and photoresponse performance of the composite. The strong absorption and excellent percolation conductive network of the Ti_3_C_2_/PEDOT:PSS composite were beneficial for the PTE effect. As a result, this PTE detector shows broadband absorption varying from 2.5 μm to 25.0 μm, with a peak detectivity of 3.5 × 10^7^ Jones at a 773 K blackbody temperature. In addition, we explored some key factors of PTE engineering, such as electrodes and substrates. The results demonstrate that the electrodes using aluminum and sputtering methods showed a higher photocurrent. The larger thermal conductivity significantly suppressed the PTE response. The device sealed in a high-vacuum environment exhibited better optical performance. To further enhance the photoresponse in this vertical PTE configuration, we fabricated gold metamaterials integrated into the detector utilizing the MXene/PEDOT:PSS composite. Finally, we proposed noncontact fingertip response applications. Although this research is only a proof-of-concept model, we foresee that this instructional material and structural engineering design will play a prominent role in wearable applications. Furthermore, PTE detectors take advantage of noncontact monitoring properties, so real-time human gestures utilizing PTE detectors can be effectively monitored. In addition, typical radiation regimes, such as terahertz radiation, exhibit nondestructive properties, and thus, future active or passive nondestructive tracking for airport security may also utilize PTE detectors.

## Materials and methods

### Materials

Ti_3_C_2_ powder was purchased from Nanochemazon. PEDOT:PSS solution (product number: 483095, 1.3 wt%, PEDOT:PSS = 5:8) and dimethyl sulfoxide (DMSO, product number: 472301) were purchased from Sigma-Aldrich®. Polyimide, conductive silver glue, and aluminum wires were purchased from Digi-Key. ITO-coated glasses and PET substrate were purchased from Hua’nan Xiangcheng Technique Company.

### Preparation of Ti_3_C_2_/PEDOT:PSS solution

First, 0.1 g of Ti_3_C_2_ powder was weighed, and the powder was added to 6.0 mL of the organic solvent DMSO. Then, the solution was agitated under magnetron stirring at 1000 rpm for 3 h, under bath sonication for 60 min, and under centrifugation at 1500 rpm for 10 min, followed by the separation of supernatant and sediment. Next, we added deionized water (DI) water and centrifuged the mixed solution, and repeated this process for four rounds. The supernatant was separated into another vial. Next, PEDOT:PSS and DMSO solution were added to the vial overnight to obtain the Ti_3_C_2_T_x_/PEDOT:PSS mixed solution, followed by magnetic stirring overnight. The composite solution remained stable for ~2 days.

### Device fabrication

ITO-coated glass substrates of 10 mm × 10 mm were cleaned by using acetone, isopropanol (IPA), and DI water in bath sonication each for 15 min, followed by N_2_ drying. Then, the glass substrates were placed on a hotplate at 100 °C for 30 min to dehydrate the surfaces. Next, we spin-coated 0.2 mL PEDOT:PSS solution on the ITO layer, followed by baking at 100 °C for 2 min. Afterward, the PEDOT:PSS membrane was dried and formed. Ti_3_C_2_T_x_/PEDOT:PSS solution (0.05 mL) was dropped onto the PEDOT:PSS layer to dry. Finally, the electrodes were prepared by the sputtering process method on the MXene/PEDOT:PSS film from Al/Au (10 nm/100 nm) layers using a shadow mask. Wires were bonded on the surfaces of ITO and gold.

### PTE detector with metamaterial fabrication

(1) A glass slide of 102 mm*83.2 mm was cleaned using a piranha wet bench (H_2_SO_4_:H_2_O_2_ = 4:1) for 15 min. DI water was used to rinse the surface and N_2_ was used for drying. (2) Acetone, IPA, and DI water were used to clean the surface, followed by hotplate prebaking at 150 °C for 10 min. (3) After the glass slide cooled down, we used an ozone system to treat the surface. (4) PMGI was spin-coated at 5000 rpm with a ramp down of 500 rpm/s, followed by Fisher oven prebaking at 150 °C for 25 min. (5) Shirpley1805 was spin-coated at 5000 rpm with a ramp down of 500 rpm/s, followed by Fisher oven prebaking at 115 °C for 25 min. (6) MLA was used for creating the metamaterials pattern with a dose of 82 mJ/cm^2^, followed by immersion in the MF-319 development solution for 70 s. (7) Then, 10 nm Ti/80 nm gold was deposited by an Angstrom E-beam, and a lift-off process was carried out using acetone with gentle sonication, followed by IPA cleaning and gentle N_2_ drying. (8) Shirpley1811 was spin-coated at 5000 rpm with a ramp down of 500 rpm/s, followed by Fisher oven prebaking at 115 °C for 25 min. This photoresist was used for creating an insulator interface to prevent the short-circuit phenomenon. (9) MLA was used to create the Al electrode pattern with a dose of 82 mJ/cm^2^, followed by immersion in the MF-319 development solution for 70 s; (10) PEDOT:PSS was spin-coated at 6000 rpm with a ramp down of 500 rpm/s, followed by baking at 30 °C for 60 min in a fume hood; (11) MXene/PEDOT:PSS solution (100 μL) was drop-casted at room temperature overnight in a fume hood. The high temperature caused the formation of bubbles between the active layer and the glass, and thus, we dried the thin film at room temperature. (12) An Al electrode of 150 nm was deposited using a PET mask fabricated by a laser cutting machine. (13) The array device was placed into acetone for 5 min, and IPA was used to clean the surface. (14) Aluminum wires were bonded to the gold and Al pad using conductive silver glue. The array circuit was completed by the laser-induced mask fabrication technique reported by ref. ^50^.

### Characterization and measurement

A scanning electron microscope (JEOL JSM-7200F SEM) at 3.0 kV voltage and 10–12 nA beam current was used to characterize the MXene/PEDOT:PSS morphology. FTIR was used for photoresponse spectrum measurement. UV‒Vis was used to measure the transparency of the MXene/PEDOT:PSS PTE detector. A blackbody radiation source (Newport Oriel 67030) was used as a photoresponse source. Keithley 6487 and Keithley 6500 were applied to characterize the current and voltage.

## Supplementary information

Supplemental Material

## Data Availability

All required data to support the results of this research work are shown in the paper and supporting information. Other data for this paper may require the submission of a request to the authors.
