# Supplementary material for: An infrared photothermoelectric detector enabled by MXene and PEDOT:PSS composite for noncontact fingertip tracking
Source: Microsyst Nanoeng. 2023 Feb 27;9:21. doi: 10.1038/s41378-022-00454-3 (PMC9968636; doi:10.1038/s41378-022-00454-3)
Supplement: Supplementary file 1 — Supplemental Material [file 41378_2022_454_MOESM1_ESM.docx]

**Supplementary Information**

**An Infrared Photothermoelectric Detector Design Enabled by MXene and Related Composite**

Jiaqi Wang^1^, Zhemiao Xie^1^, Guanxuan Lu^1^, Jiayu Alexander Liu^1^, John T.W. Yeow^1,*^

^1,*^ Advanced Micro-/Nano- Devices Lab, Department of Systems Design Engineering, University of Waterloo, 200 University Ave West, Waterloo, Ontario, N2L 3G1, Canada

Corresponding Author: John T.W. Yeow

E-mail: [jyeow@uwaterloo.ca](mailto:jyeow@uwaterloo.ca)

Phone and fax: +1-519-888-4567, ext.32152


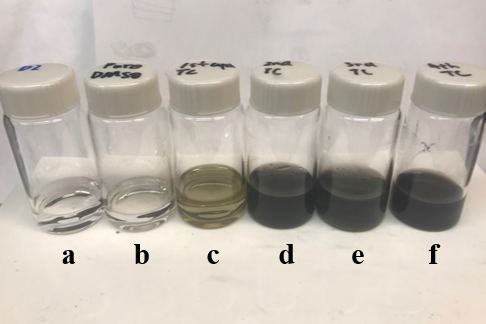


**Fig. S1:** A comparison of pristine Ti_3_C_2_ solution by adding DI water removing the supernatant solution. **a** DI water. **b** DMSO. **c**, **d**, **e**, and **f** are the 1^st^, 2^nd^, 3^rd^, and 4^th^ cycled Ti_3_C_2_ dispersion solution by removing the supernatant and adding DI water.


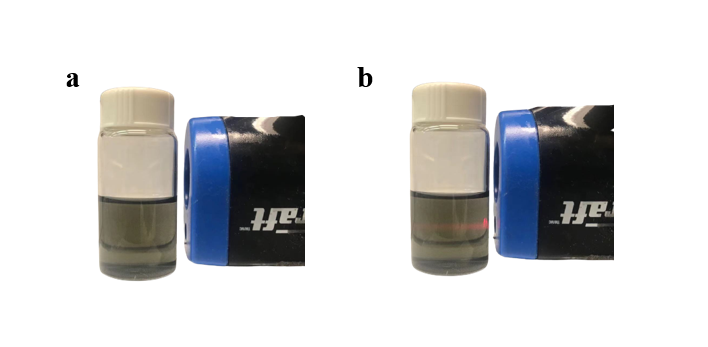


**Fig. S2:** Ti_3_C_2_ dispersed in **a** DMSO and **b** DI water solution. The Tyndall effect can be clearly seen in DI water dispersion. The spectrum of the laser is 630-670 nm.


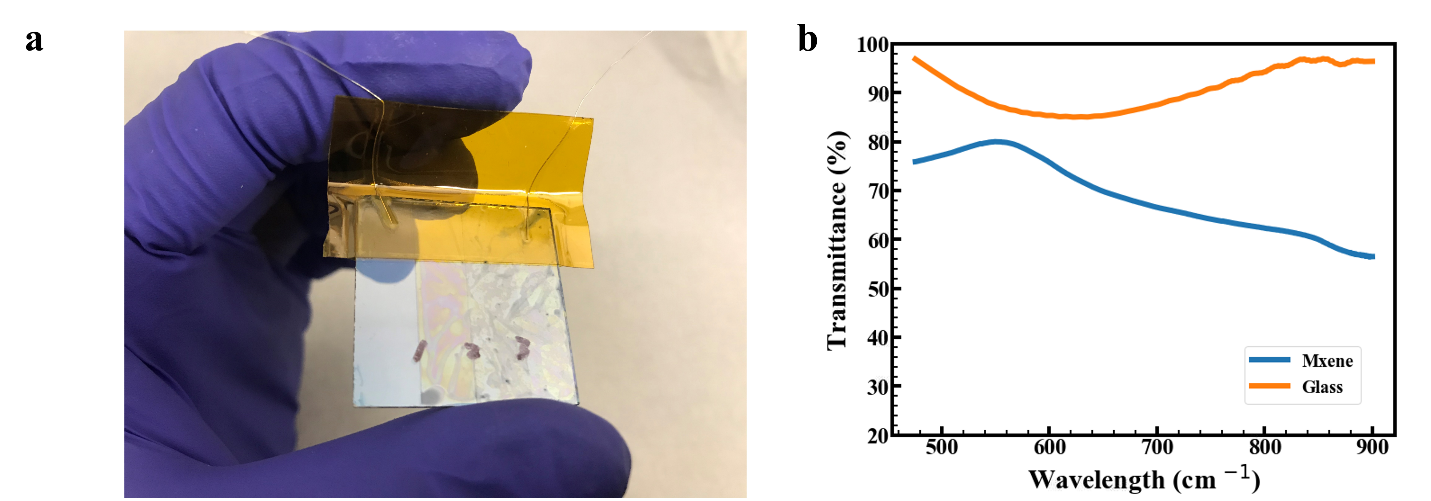


**Fig. S3: a** Photographic imaging of semi-transparent PTE detector based on ITO/(Ti_3_C_2_/PEDOT:PSS)@PEDOT:PSS/ITO. **b** UV-Vis spectrum during 475-900nm regime.

**
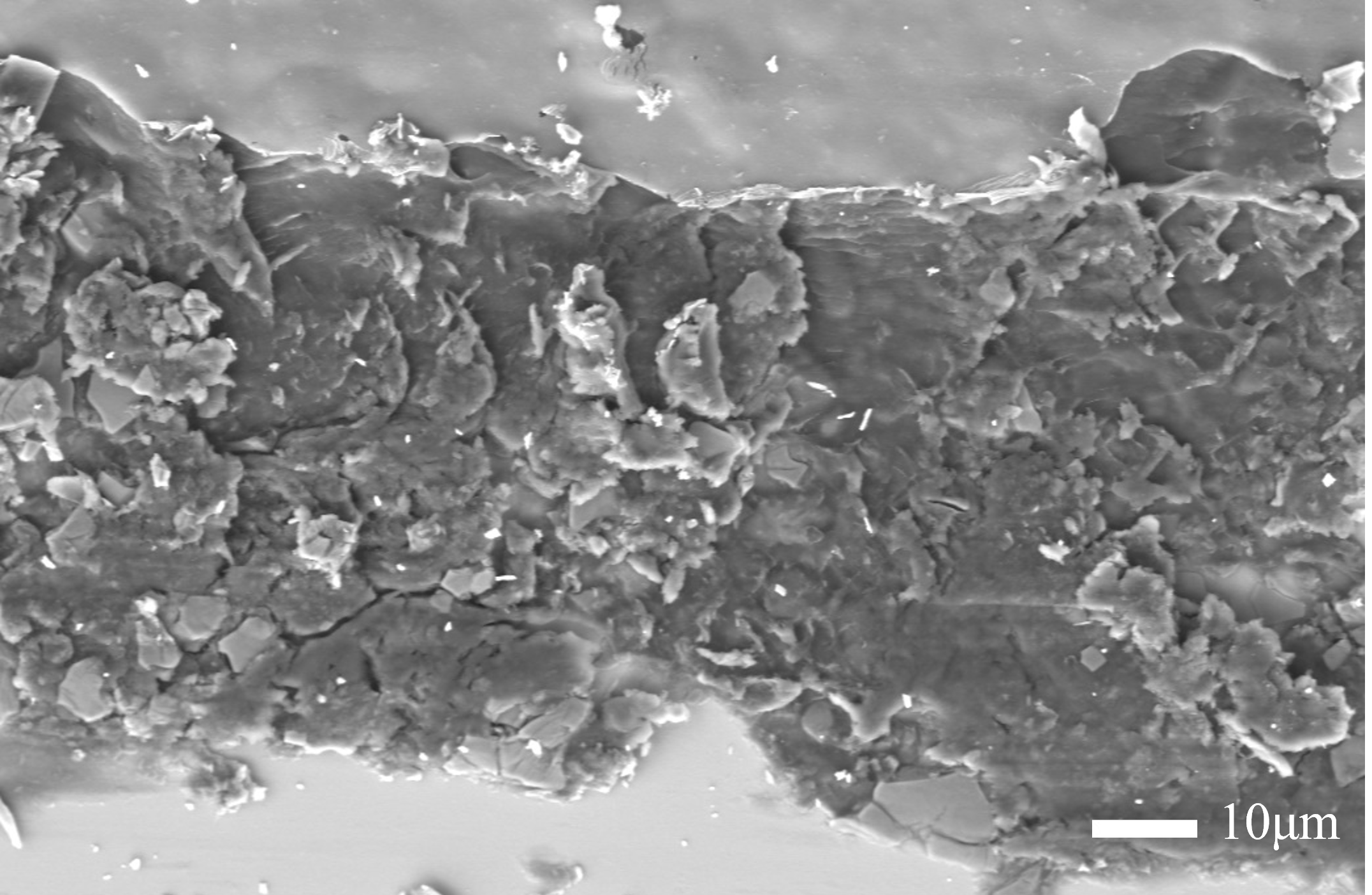
**

**Fig. S4:** MXene flake pieces.

**
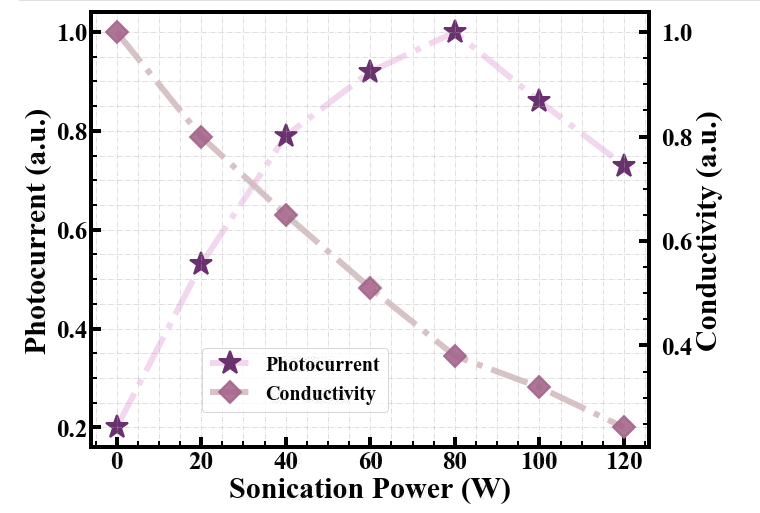
**

**Fig. S5:** The photoreponse and conductivity dependence on sonication power.


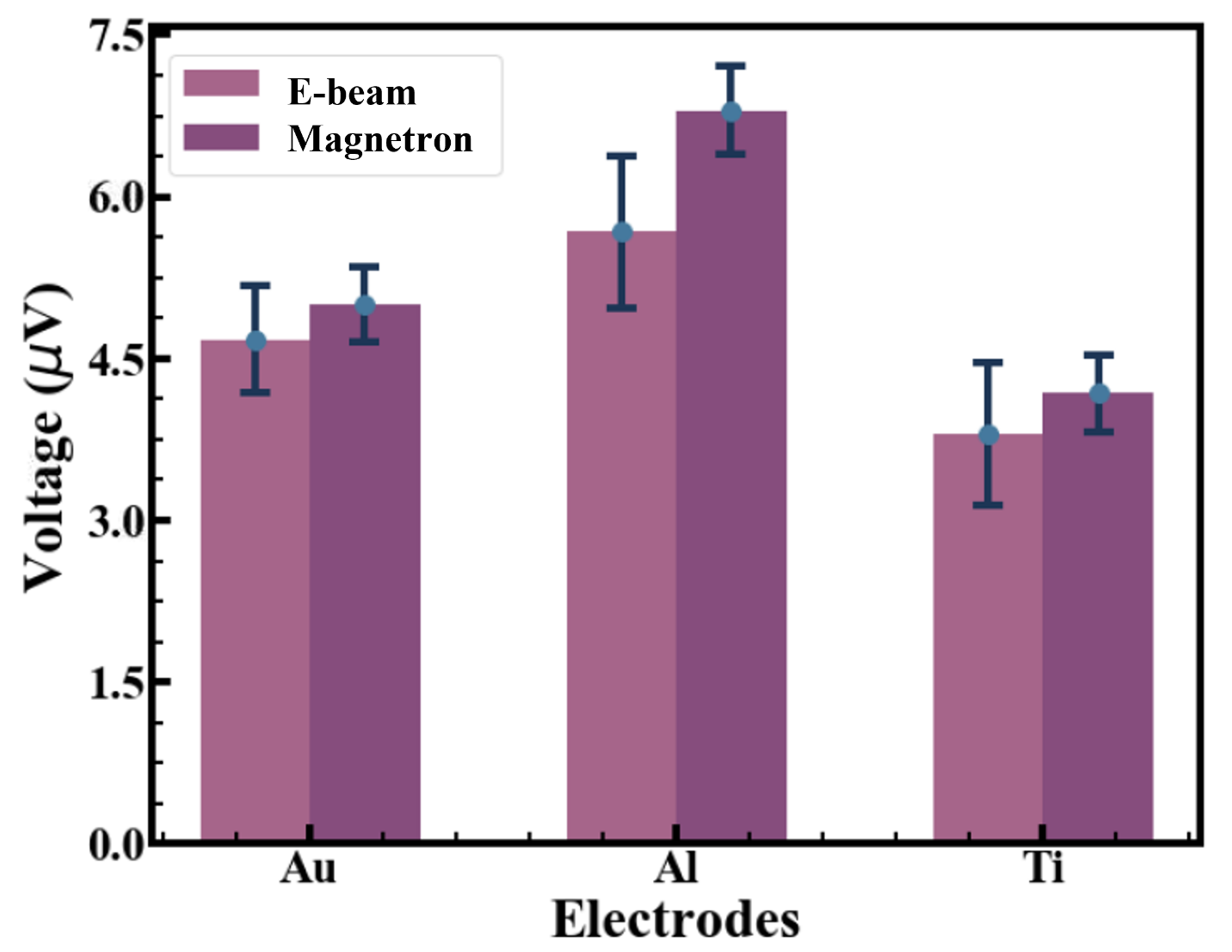


**Fig. S6:** The photovoltage changes with different electrodes using E-beam or magnetron.


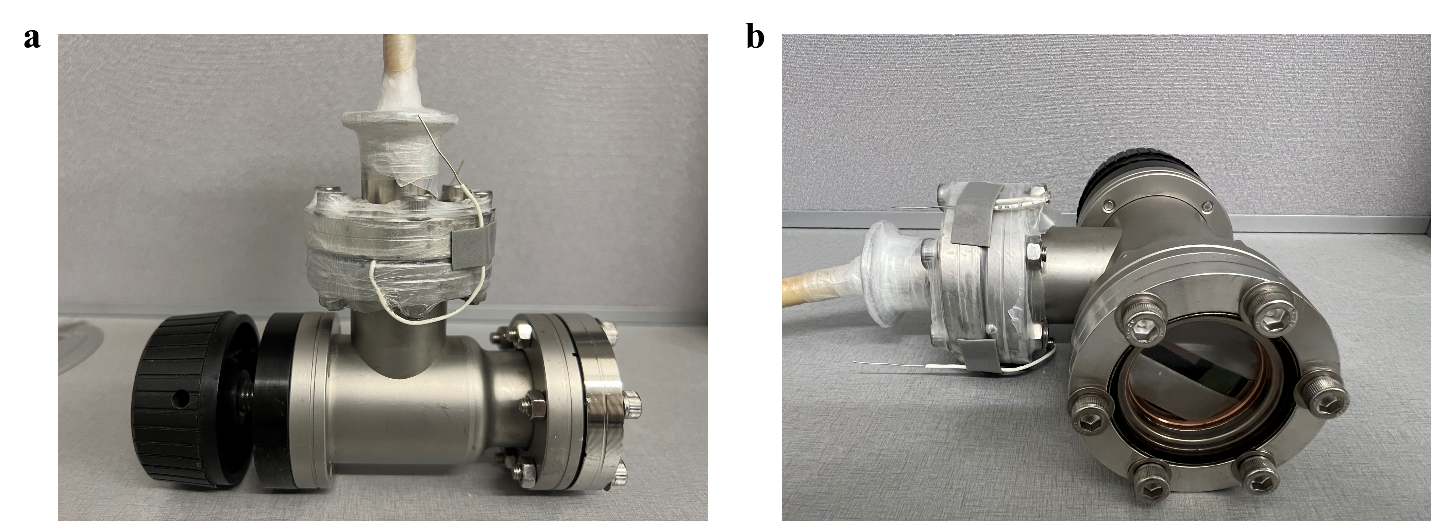


**Fig. S7:** The optical imaging of PTE detector under vacuum condition.


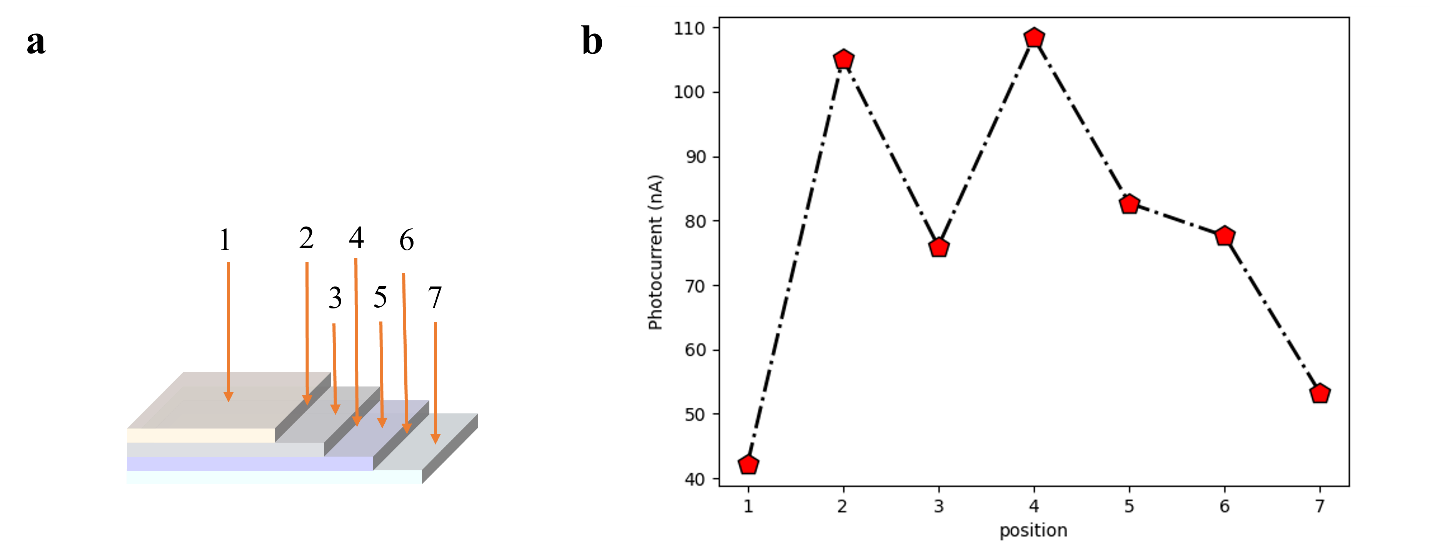


**Fig. S8: a** The irradiation labels in the device. **b** The photocurrent dependence on irradiation position.

***
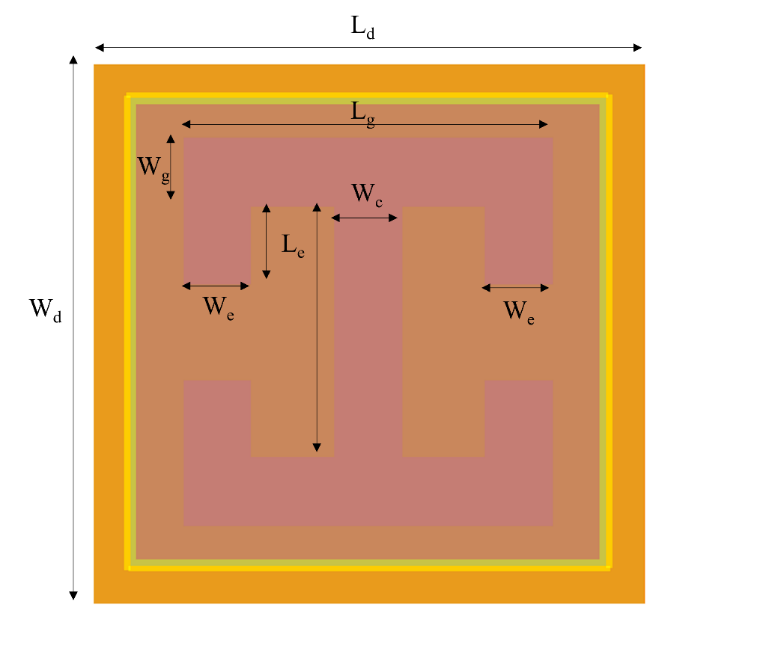
***

**Fig. S9:** The illustration of one metamaterial unit.


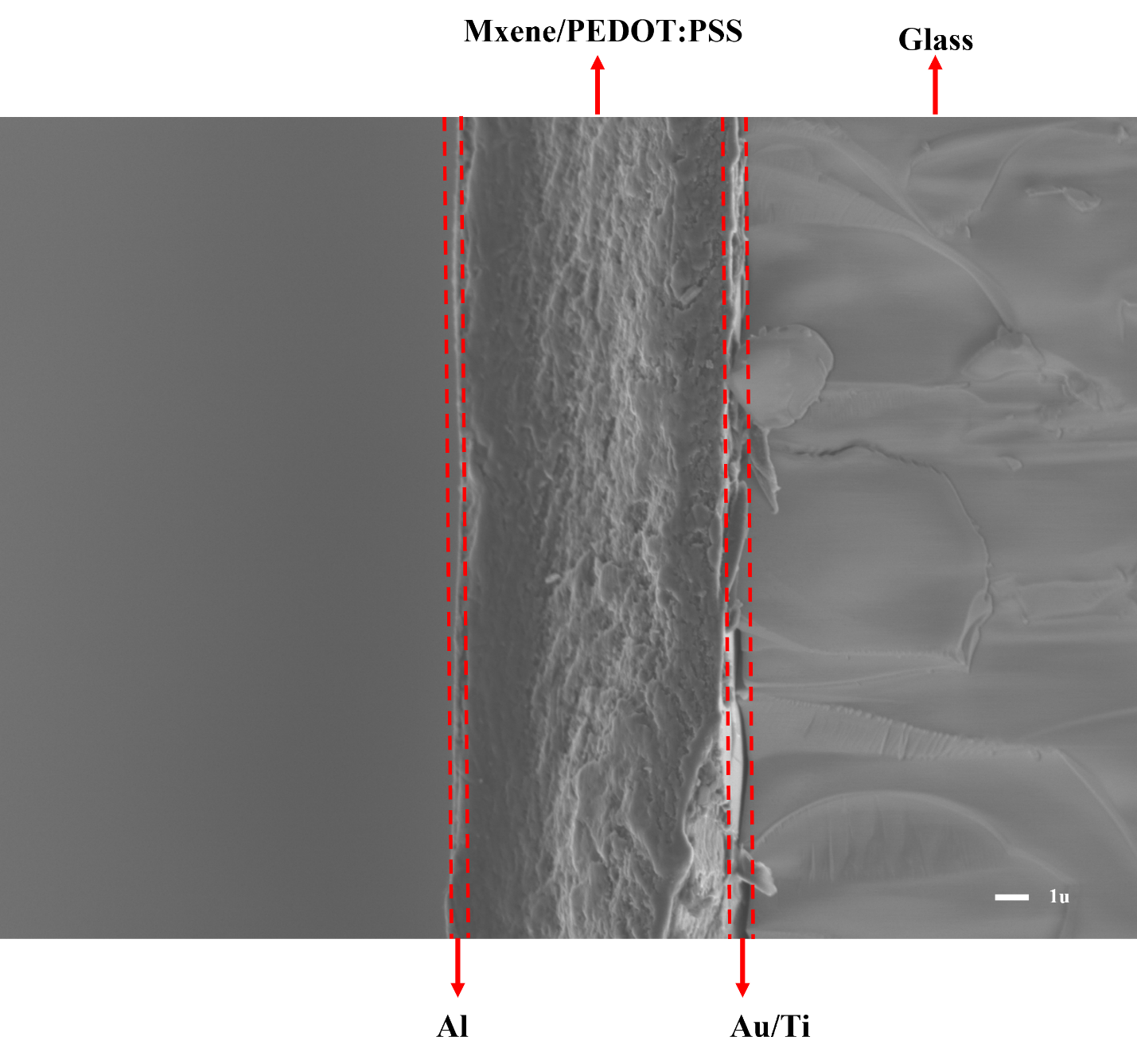


**Fig. S10:** The cross-sectional SEM of one as-fabricated metamaterial PTE detector.


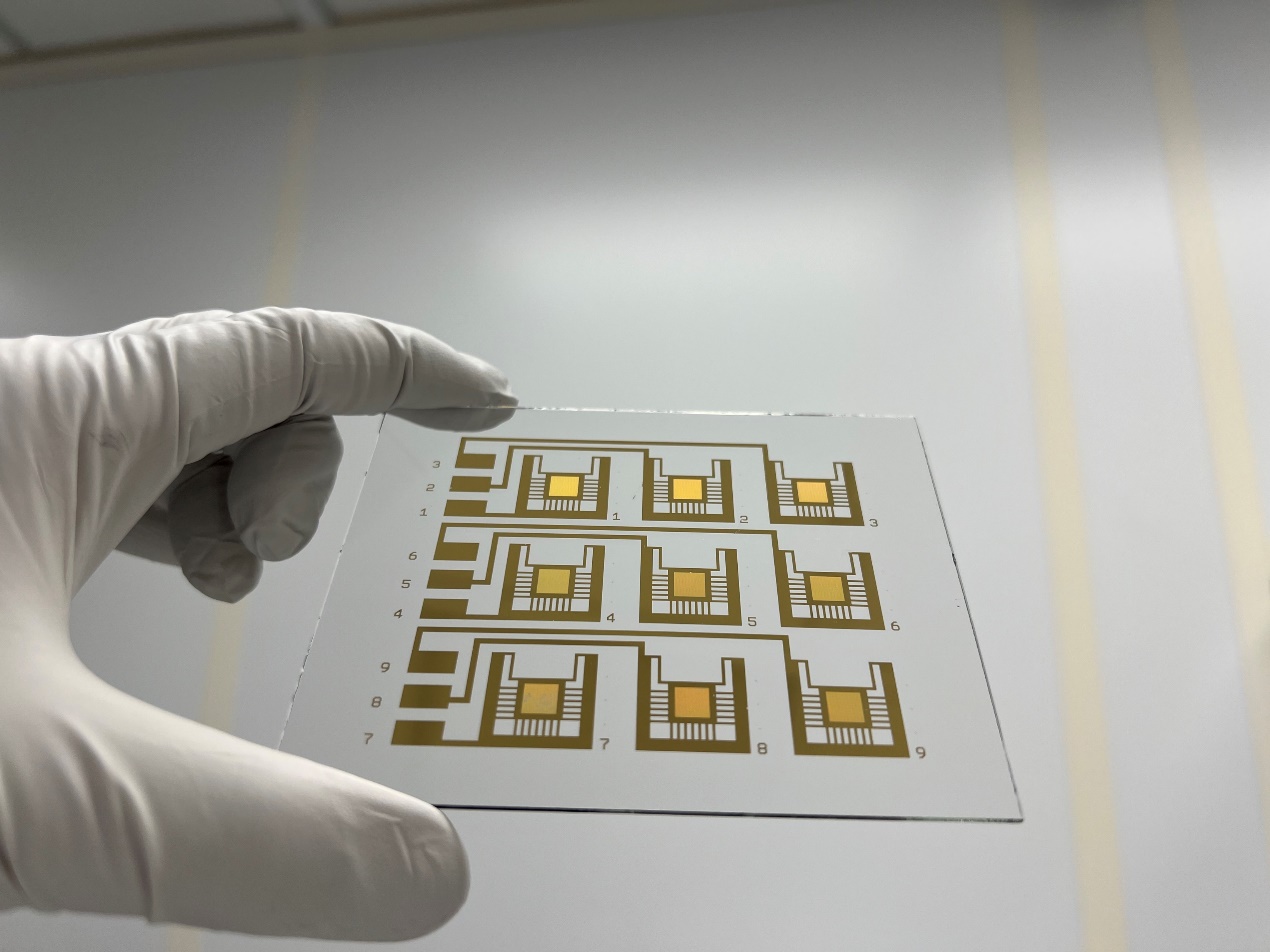


**Fig. S11:** The front optical imaging of metamaterial structure.

**
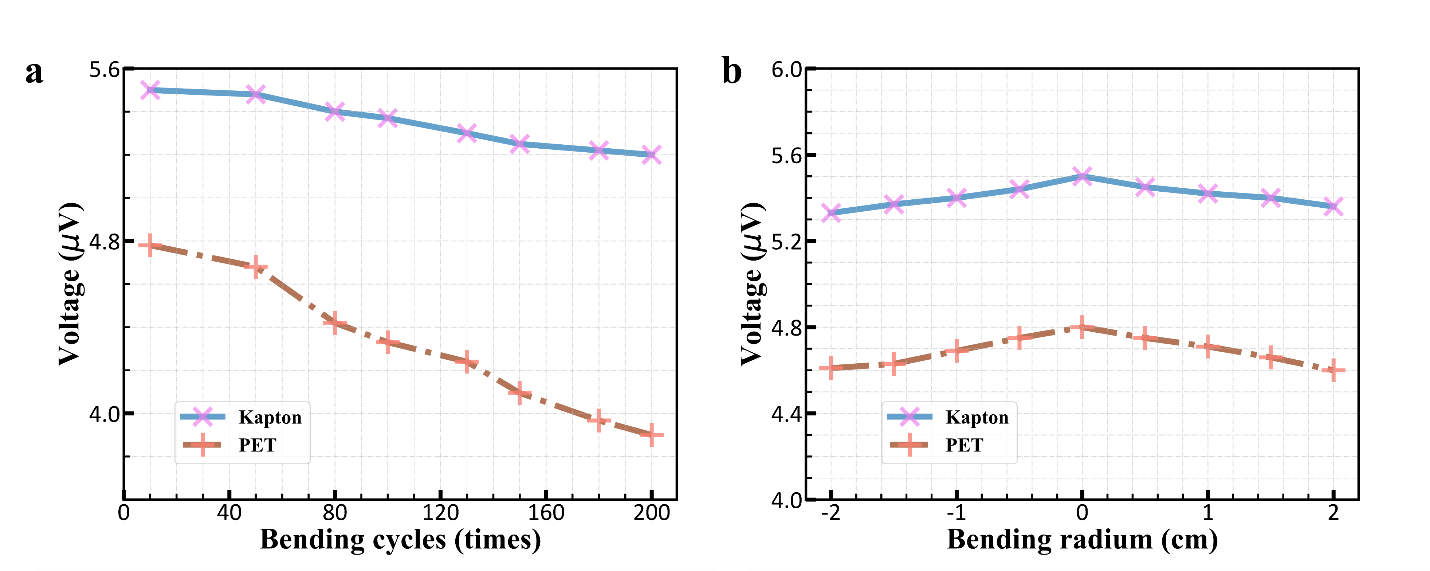
**

**Fig. S12: a** The photovoltage changes with different **a** bending cycles and **b** bending radium**.**

**Table S1:** The design of one metamaterial unit.

| Dimensions | Value (μm) |
| --- | --- |
| Device length, L_d_ | 5.0 |
| Device width, W_d_ | 5.0 |
| Thickness of below gold layer | 0.080 |
| Thickness of MXene layer | 50 |
| Thickness of upper gold layer | 100 |
| Length of the etching capacitor gap, L_g_ | 8.0 |
| Width of the etching capacitor gap,W_g_ | 1.5 |
| Channel length, L_c_ | 5.5 |
| Channel width, W_c_ | 1.5 |
| Thickness of the channel | 110 |
| Thickness of titanium layer | 10 |
| Thickness of top glass layer | 3000 |
| Length of extended etching capacitor gap, L_e_ | 1.7 |
| Width of extended etching capacitor gap, W_e_ | 1.5 |

**Note:**

The MXene simulation data is from Ref. ^1^.

**Reference**

1. Miranda, A., Halim, J., Lorke, A. & Barsoum, M. W. Rendering Ti_3_C_2_T_x_ (MXene) monolayers visible. *Mater. Res. Lett.* **5**, 322–328 (2017).
